# The Management of Patients at High Risk of Serious COVID-19 Disease: Optimising the Patient Pathway in the Middle East, Africa, and Eastern Europe

**DOI:** 10.7759/cureus.60727

**Published:** 2024-05-20

**Authors:** Jameela Al-Salman, Ashraf Amir, Luke SP Moore, Garyphallia Poulakou, Alex Soriano, Jehad Abdallah, Akaki Abutidze, Wagdy Amin, Gehan El Assal, Magula Nombulelo, Feras Tarawneh, Ashraf Hassanien

**Affiliations:** 1 Medicine, Arabian Gulf University, Manama, BHR; 2 Executive Medical Services, International Medical Center Hospital, Jeddah, SAU; 3 Infectious Diseases, Chelsea and Westminster NHS Foundation Trust, London, GBR; 4 Internal Medicine, National and Kapodistrian University of Athens, Sotiria General Hospital, Athens, GRC; 5 Infectious Diseases, Hospital Clínic de Barcelona, Barcelona, ESP; 6 Infectious Diseases, Al Rahba Hospital, Abu Dhabi, ARE; 7 Infectious Diseases, AIDS and Clinical Immunology Research Center, Tbilisi, GEO; 8 Chest Diseases, Ministry of Health and Population, Cairo, EGY; 9 Chest Diseases, Ain Shams University, Cairo, EGY; 10 Internal Medicine, University of KwaZulu-Natal, Durban, ZAF; 11 Internal Medicine, Tarawneh Clinic, Amman, JOR; 12 Medical affairs, Pfizer Ltd., Jeddah, SAU

**Keywords:** unmet needs, antivirals, access to treatment, high-risk patients, covid-19

## Abstract

Introduction

For patients at high risk of severe COVID-19 disease, antiviral therapeutic options are available to reduce the risk of hospitalization or death. Although many countries have developed national guidelines for COVID-19 management that include use of antiviral agents, it is unclear how these guidelines are used in daily clinical practice. This study aims to assess the management of high-risk COVID-19 patients in the Middle East, Africa, and Eastern Europe, with a focus on understanding current practices, challenges, and potential strategies for improvement.

Methods

Healthcare professionals (HCPs) from the Middle East, Africa, and Eastern Europe came together at a regional summit in February 2023 to share perspectives on the therapeutic management of patients at high risk of serious COVID-19 disease in the community. Summit participants represented diverse medical specialties, geographical regions, and healthcare settings. Key insights gathered during the summit were supplemented with evidence from the published literature via a non-systematic literature search of MEDLINE and online sources such as government reports since the start of 2020 to identify articles on disease burden, unmet needs, treatment access, antiviral therapy, guidelines related to individuals with COVID-19 at high-risk for poor outcomes in low- and middle-income countries (LMICs). Together, these sources were used by the authors to generate their recommendations for future priorities and optimal care pathways globally.

Results

Specific insights gathered from the summit were that participants reported that primary care is the first point of contact for high-risk patients, but the role of primary care physicians (PCPs) in treatment is uncertain. Additionally, participants highlighted that between-country differences in the care pathway for high-risk patients are due to variations in local treatment practices, healthcare system structures, and resourcing. In line with the published literature, participants agreed that HCP education is needed to support the identification, counseling, and appropriate management of high-risk patients and that pharmacists have a critical role to play in identifying clinically important potential interactions with antiviral treatment and recommending appropriate adjustments. Furthermore, patient hesitancy can result in late presentation, delayed treatment, and potential progression of symptoms. HCPs should proactively counsel high-risk patients, so they are aware of their risk and its implications and understand what to do if they experience symptoms of COVID-19. Targeted educational initiatives for patients are needed to mitigate reluctance to undergo COVID-19 testing and counter COVID-19 misinformation.

Conclusion

Collaboration among stakeholders is essential to optimize COVID-19 management for high-risk patients globally, ensuring effective implementation of guidelines and improving outcomes.

## Introduction

COVID-19 infection continues to result in significant mortality and morbidity [1]. Globally, a total of 774,954,393 cases of COVID-19 have been reported, and 7,040,264 deaths occurred due to the disease as of March 2024 [2]. A number of studies have reported the significant burden of COVID-19 disease in the Middle East and Africa, particularly among older patients and those with comorbidities that elevate their risk of experiencing serious COVID-19 disease [3-5]. For example, a study conducted in the Kingdom of Saudi Arabia (KSA) confirmed that COVID-19 patients with comorbidities such as lung disease, higher body mass index (BMI), smoking, kidney disease, and diabetes mellitus had the highest risk for intensive care unit (ICU) admission [3]. Higher BMI and co-existing diabetes mellitus or kidney disease were also identified as risk factors for ICU admission in COVID-19 patients in Qatar [6], while in Bahrain, the risk of ICU admission was highest in those with immunocompromised conditions, lung disease, and hypertension [5]. In African countries, comorbid human immunodeficiency virus/acquired immunodeficiency syndrome (HIV/AIDS), diabetes mellitus, liver disease, and kidney disease were associated with increased mortality in COVID-19 patients [4].

For patients at high risk of progression to severe disease, antiviral therapeutic options are available to reduce the risk of hospitalization or death. However, there is a lack of understanding and alignment on the role of antiviral therapy in high-risk COVID-19 patients, criteria, definitions, and recognition of high-risk patients, the eligibility criteria and key considerations for antiviral therapy, and awareness of clinical trials and real-world experience evidencing best practice approaches for managing such patients.

Although many countries in the Middle East and Africa have developed national guidelines for the management of COVID-19 that include the use of antiviral agents, it is unclear how these guidelines are used in daily clinical practice across these regions. The World Health Organization (WHO) has highlighted examples of successful COVID-19 preparedness, partnership, and engagement in healthcare systems in the region, such as in Bahrain [7]. However, a review that evaluated the impact of acute care interventions on clinical outcomes in individuals with severe acute respiratory infections (SARIs) found that a lack of evidence exists for best practices in the management of SARIs in low- and middle-income countries (LMICs) [8].

This study aims to 1) assess the current management practices for high-risk patients with COVID-19 in the Middle East, Africa, and Eastern Europe; 2) identify barriers and challenges faced in the management of high-risk COVID-19 patients across different regions; 3) explore the roles of primary care providers (PCPs) and pharmacists in the patient pathway for high-risk COVID-19 cases; 4) provide recommendations for optimizing the patient pathway and improving outcomes for high-risk COVID-19 patients in diverse healthcare settings; 5) enhance understanding of the global perspective on antiviral therapy utilization and adherence to guidelines among healthcare professionals.

A regional summit was held with healthcare professionals (HCPs) experts from the Middle East, Africa, and Eastern Europe countries. During the summit, participants reached a consensus on the current management practices and future directions for COVID-19 among high-risk patients. A literature search on the practices for COVID-19 management was performed to support the overall objective of this article, which is to bring together the diverse perspectives from across the region, to learn from the diverse local responses and experiences, and align on optimal clinical pathways for the management of high-risk patients as we move into a post-pandemic era of COVID-19 care.

## Materials and methods

A regional two-day summit was held in Dubai, United Arab Emirates (UAE) during February 2023, involving HCPs from the Middle East, Africa, and Eastern Europe. The objective of the summit was to collect qualitative insights on how COVID-19 management guidelines translate into real-world clinical practice and how clinical practice can be optimized going forward toward optimal management of high-risk COVID-19 patients. A greater understanding of current practices and aspirations for optimal management will inform ongoing discussions about future pandemic readiness. The summit was a face-to-face meeting moderated by a faculty of six international and regional experts. Given the pressing need to reduce serious illness in high-risk patients, two key focus areas for investigation and alignment with participants were 1) the need to better understand the current situation in their respective countries and healthcare systems regarding the treatment journeys of high-risk patients with COVID-19, local management guidelines, and barriers to access to antiviral treatments; and 2) the need to align on the optimal care pathway for high-risk patients with COVID-19, including creation of the appropriate infrastructure, planning for HCP training/education, and communication initiatives to increase patient and public awareness.

Snowball recruitment was used to identify eligible participants [9]. To be eligible, individuals needed to be practicing HCPs caring for high-risk patients with COVID-19. Country representatives nominated eligible HCPs, and a total of 40 HCPs from different medical specialties, a range of geographical regions, and different healthcare settings from Bahrain, Egypt, Georgia, Ghana, Jordan, KSA, Kuwait, Morocco, Oman, South Africa, and UAE attended the summit. Participants were infectious disease, primary care, family medicine, and emergency department physicians, and clinical pharmacists, i.e., all were front-line HCPs likely to be who a high-risk patient would present to with suspected COVID-19 infection or symptoms. Participants' practice settings represented the range of healthcare settings in the region, including private clinics, public/national health systems, Ministries of Health and military hospitals, and academic institutions/tertiary healthcare settings. Some participants were also members of COVID-19 advisory committees in their countries. Formal consent for publishing the participants' insights and collected data was obtained from all participants.

The summit agenda was designed to facilitate experience-sharing and brainstorming, with workshops to allow all participants to share their views, clinical experiences, and healthcare system structures. Participants were divided into three working groups, and each of them discussed the themes "enabling infrastructure for adequate care of patients at high risk for COVID-19", "HCP and pharmacist preparedness to deal with patients at high risk for COVID-19", and "patient and public awareness of patients at high risk for COVID-19". Each working group was composed of 12 or 14 participants representing a range of different countries, and the discussions were facilitated by the authors of this manuscript. Flipcharts were used to support the discussion. The workshops were recorded, and the discussion transcripts were used for data analyses. Similar ideas from the three working groups were categorized into statements that represented the consensus of the whole group.

With regards to treatment, nirmatrelvir-ritonavir was the antiviral therapy of focus, as it is the currently preferred antiviral agent for treatment of ambulatory high-risk patients with non-severe COVID-19 in major treatment guidelines [10, 11] and had received emergency use authorisation in all of the meeting participants' countries during the COVID-19 pandemic.

Key insights gathered from summit participants were considered by the authors in the context of published biomedical literature sourced via a non-systematic literature search of MEDLINE and online sources such as government reports since the start of 2020 to identify articles on disease burden, unmet needs, treatment access, antiviral therapy, guidelines related to individuals with COVID-19 at high-risk for poor outcomes in LMICs. Together, these sources were used by the authors to generate their recommendations for future priorities and optimal care pathways globally.

## Results

Current treatment pathways for high-risk patients with COVID-19

Primary Care Is the First Point of Contact for Patients

Primary care providers are generally the first line of care in the community, maintaining continuity of care for people with chronic conditions and reducing pressure on hospital services [12,13]. Primary care physicians (PCPs) are optimally placed to identify which patients are at high risk of serious COVID-19 disease and to manage testing and triage care should their high-risk patients become infected.

Participants from Georgia, Morocco, Jordan, KSA, UAE, Bahrain, Kuwait, and Oman reported that high-risk outpatients with COVID-19 are most likely to be seen by PCPs, whereas in South Africa and Ghana, they are more likely to be seen by nurses and community support workers.

In all countries represented at the summit, the main role of community pharmacists currently is to dispense medication and sometimes to provide private COVID-19 testing and vaccination. In the United States (US), the role of pharmacists has been extended to prescribing nirmatrelvir-ritonavir for high-risk patients with COVID-19, albeit under certain conditions [14]. Benefits of this approach include improving access in communities with fewer physicians and expanding access to timely treatment, given that nirmatrelvir-ritonavir must be taken within five days of symptom onset.

Definitions of High-Risk Populations Vary Globally

COVID-19 patients who have the highest risk of hospitalization typically include unvaccinated individuals, older people, and those with compromised immune systems or comorbid chronic diseases [10]. Except for Ghana and South Africa, all participant countries had either developed their own formal definition of a high-risk COVID-19 patient (described in Ministry of Health guidelines) or used the definition of an international body, such as the WHO [15] (Table 1).

**Table 1:** WHO definition of risk factors associated with high risk of severe COVID-19 disease HIV - human immunodeficiency virus; WHO - World Health Organization

Risk factors associated with severe COVID-19 disease
Age >60 years (increasing with age)
Underlying noncommunicable diseases such as diabetes, hypertension, cardiac disease, chronic lung disease, cerebrovascular disease, dementia, mental disorders, chronic kidney disease, immunosuppression, obesity, and cancer
Pregnant or recently pregnant women aged >35 years, with obesity or a chronic medical or pregnancy-specific condition (such as gestational diabetes or pre-eclampsia/eclampsia)
Smoking
Unvaccinated against COVID-19
HIV

All participants agreed that many high-risk patients, especially those with lower education levels, do not recognize that they may be at high risk of hospitalization, and those who received COVID-19 vaccination may perceive their risk to be lower. However, immunocompromised patients generally have a high level of understanding of their risk.

Timely Patient Presentation and Testing Is a Challenge in Some Countries

Participants reported that rapid antigen testing (RATs) is readily available, polymerase chain reaction (PCR) testing is accessible in most countries, and generally diagnostic tests are partially or fully funded. The exception was in African countries, where there is no self-testing and RATs are administered by HCPs.

However, the time typically taken for high-risk patients to seek medical help after developing symptoms or testing positive for COVID-19 varied between countries. Nirmatrelvir-ritonavir must be taken within five days of symptom onset [16], and COVID testing is a prerequisite for its use [16,17], so some resource-constrained countries might struggle to diagnose and treat cases quickly enough within this treatment window [17,18]. Participants reported that in Bahrain, Kuwait, Morocco, Oman, and UAE, patients typically seek help after one to two days, whereas patients in Georgia and Jordan often wait five to six days, those in KSA wait three to five days, and those in Egypt three to seven days. In Ghana and South Africa, high-risk patients test only when very sick (for example, requiring oxygen) and present very late. Patients in Bahrain, Egypt, Jordan, Kuwait, Oman, and UAE typically self-treat their symptoms before seeking medical services.

These data indicate that in many countries, there is a high likelihood of patients being ineligible for antiviral treatment, and therefore, there is a need to address the problem of late patient presentation.

The Typical Patient Journey Across Different Countries Today

**Figure 1:**
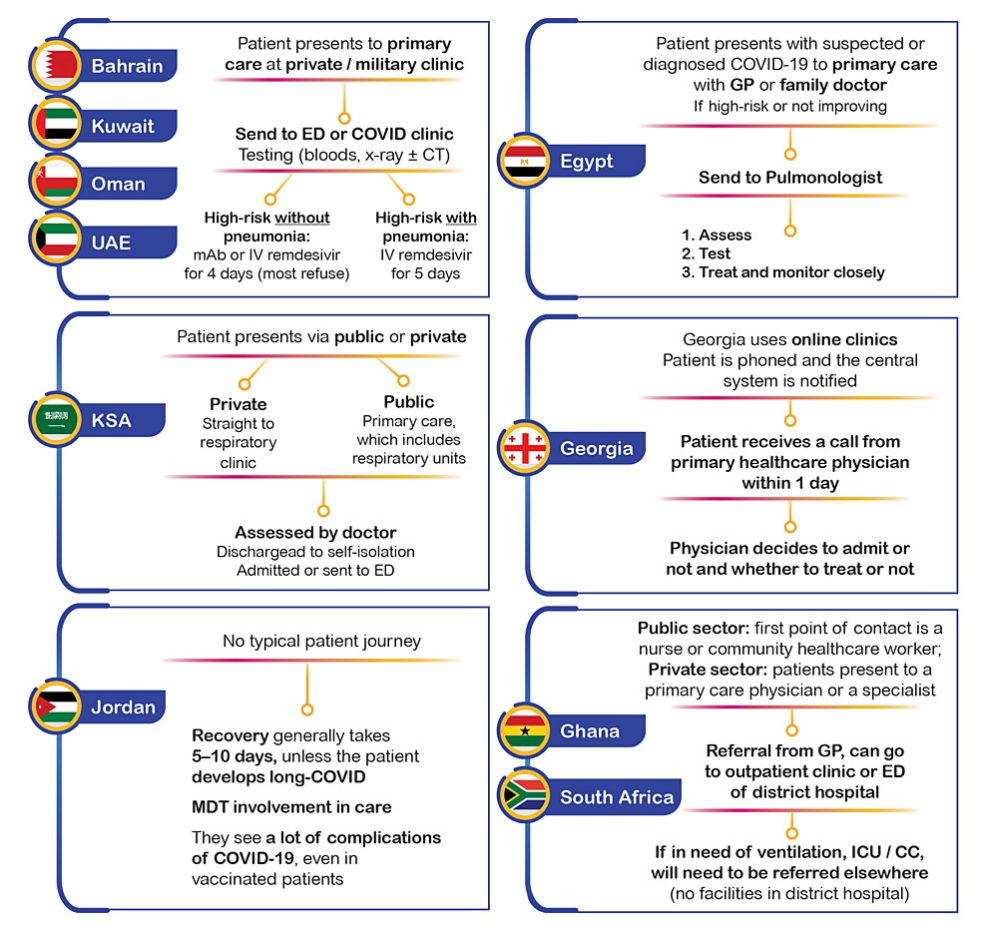
The typical patient journey for high-risk patients with COVID-19 across different countries of the Middle East, Africa, and Eastern European regions at the time of the summit CT - computed tomography; ED - emergency department; GP - general practitioner; ICU / CC - intensive care unit / critical care; IV - intravenous; KSA - Kingdom of Saudi Arabia; mAb - monoclonal antibody; MDT - multidisciplinary team; UAE - United Arab Emirates

The typical patient treatment journey varies significantly in complexity between countries. The differences in patient journeys are primarily a reflection of differently resourced and structured healthcare systems and variations in the practice guidelines for high-risk COVID-19 patients used in the different countries (Figure 1).

Lack of Consistency Across National Guidelines for Management of High-Risk Patients

All the countries represented have specific guidelines for the management of high-risk COVID-19 patients, although those in Ghana and South Africa were considered to lack clear guidance. Only the guidelines in Bahrain, Georgia, KSA, Kuwait, Oman, and UAE included recommendations on the use of nirmatrelvir-ritonavir.

Perceived Barriers to Antiviral Treatment

Early administration of oral nirmatrelvir-ritonavir has been demonstrated to reduce the risk of emergency department visits, hospitalization, and death in community-living individuals (including individuals at high risk of severe disease) with early symptomatic COVID-19 in clinical trial and real-world settings [2,19,20]. As an effective oral antiviral agent that can be administered at home [16], nirmatrelvir-ritonavir treatment places minimal demand on HCPs and reduces the need for oxygen support, which may be an advantage in countries with resource-limited healthcare systems [17].

From patient and HCP perspectives, participants identified the main barriers to treatment of high-risk COVID-19 patients with nirmatrelvir-ritonavir as 1) patients presenting more than five days after symptom onset; 2) physician anxiety around managing comedications and potential concomitant medication interactions with nirmatrelvir-ritonavir; 3) inability of patients to pay if the drug is not funded/reimbursed; and 4) lack of data on the use of nirmatrelvir-ritonavir in patients with HIV, which is particularly relevant to African countries with high rates of HIV infection.

Healthcare system priorities for effective management of high-risk patients

Patient Reluctance to Test May Compromise Antiviral Treatment

Participants agreed that globally, the general population is becoming increasingly reluctant to undergo testing for COVID-19, given that a positive test may lead to mandatory isolation, employment issues, hospitalization, travel restrictions, and other personal challenges. The consequence of this reluctance is often delayed diagnosis and presentation; this delay can have serious consequences for high-risk patients who may need antiviral treatment within a specific time period (for example, the five-day treatment window for nirmatrelvir-ritonavir).

Recommendation one: Primary care HCPs should partner with other stakeholders to mitigate the lack of testing, for example, allowing empiric antiviral treatment prior to laboratory confirmation (as is done for certain high-priority patients with suspected influenza), facilitating community access to RATs, promoting the acceptability of RATs as an alternative to PCR testing, and building local awareness on the importance of testing in high-risk patients.

Comedications Are a Concern With Antiviral Treatments, but Prescribers Can Manage Risks

Nirmatrelvir-ritonavir can interact with other commonly prescribed medications, particularly with drugs that are strong CYP3A inducers or highly dependent on CYP3A for clearance [16]. Comedications are a particularly important consideration in high-risk COVID-19 patients, who often have a range of comorbid health conditions and may be receiving numerous comedications, such as statins, calcium channel blockers, and direct oral anticoagulants [9]. Furthermore, data are lacking on the magnitude of risk and severity of potential drug-drug interactions (DDIs) that may occur from the use of ritonavir-containing COVID-19 therapy in the general population [21].

Recommendation two:** **With access to education and resources, such as peer-to-peer knowledge exchange initiatives and the University of Liverpool's online drug interactions tool [22], prescribers can gain confidence to manage the potential for DDIs and optimize outcomes for high-risk patients. Consideration should be given to producing physical resources for use in countries where access to electronic or web-based resources may be limited.

The Role of Primary Care Varies Globally

There was broad agreement that PCPs play a vital role in triaging high-risk COVID-19 patients and referring them to a specialist or emergency care. In some countries (such as Jordan), additional value was perceived for high-risk patients with COVID-19 when they were both identified and then prescribed antiviral treatment in the primary care setting. In other countries (such as Egypt), this approach was not felt to be appropriate.

Recommendation three:** **PCPs would benefit from additional education and training to improve their confidence and capabilities in the management of high-risk patients, particularly in countries where this is appropriate within the existing structure of the healthcare system. Existing primary care educational programs could be leveraged from countries where PCPs already play a significant role in the management of patients at high risk of progression to severe disease.

Pharmacists Provide Valuable Support but Are Not Prescribers

Community pharmacists are trusted HCPs and readily accessible to patients [23,24]. Management of both testing and treatment of high-risk patients by pharmacists as a single point of care could be a potential strategy for reducing barriers to accessing antiviral therapies for COVID-19 during times of increased need in the community and pressure on healthcare systems [23].

Participants agreed that pharmacists play an integral role in screening for medications with potential interactions with antiviral treatments and flagging the need for dosage adjustment. However, there was limited support for expanding the role of pharmacists to become prescribers of antiviral therapies directly.

Recommendation four:** **Tailored education for pharmacists that is relevant to their role(s) in the treatment pathway for high-risk patients would be valuable (such as on screening for potential DDIs and recommending mitigation strategies, identifying the need for dosage adjustment for special populations such as patients with liver or renal impairment).

Limited Data in Patients With HIV May Have Led to Hesitancy in Some Countries

Guideline recommendations for the diagnosis and treatment of COVID-19 among people with HIV are the same as those for the general population [9]. People living with HIV appear to have the same risk of mortality from COVID-19 as the general population, although data from sub-Saharan Africa, where HIV is highly prevalent, are limited [25].

Participants believed that limited data on the use of nirmatrelvir-ritonavir in patients with HIV may be a source of hesitancy among physicians in countries with high rates of HIV infection, such as South Africa. This concern is understandable in light of an analysis that an estimated 33% of the US population would be at risk for a potential DDI should they receive a ritonavir-containing COVID-19 therapy, with the risk increasing significantly in those aged ≥60 years and with comorbidities including HIV [21].

Recommendation five: There is a need for greater sharing of experience (such as via peer-to-peer knowledge-sharing initiatives) between physicians who have treated HIV-positive patients with nirmatrelvir-ritonavir successfully and those who may be hesitant to do so. Data generation on nirmatrelvir-ritonavir use in HIV patients (such as post hoc sub-group analyses of clinical trials and real-world studies in high-prevalence countries) would be highly valuable.

Overcoming barriers and delivering solutions for optimal care

During the summit, participants worked collaboratively to develop: 1) a patient pathway that would create an enabling infrastructure for best-practice management of high-risk patients with COVID-19 that could be adopted globally; 2) the ideal communication and training/education for HCPs to ensure high-risk patients are identified and management appropriately; and 3) strategies for raising patient and public awareness of the ongoing risks of COVID-19 for some members of the community and how these risks can be managed effectively.

Enabling Infrastructure

Participants agreed that effective communication prior to infection is critical to facilitate effective patient identification and timely treatment. Recommended actions included local education programs targeted at the general population, potential high-risk patients, and primary care teams focused on raising awareness of the risks of COVID-19; development and implementation of guidelines focusing on the treatment of high-risk patients with COVID-19; and proactive identification of those at high risk of serious COVID disease.

**Figure 2:**
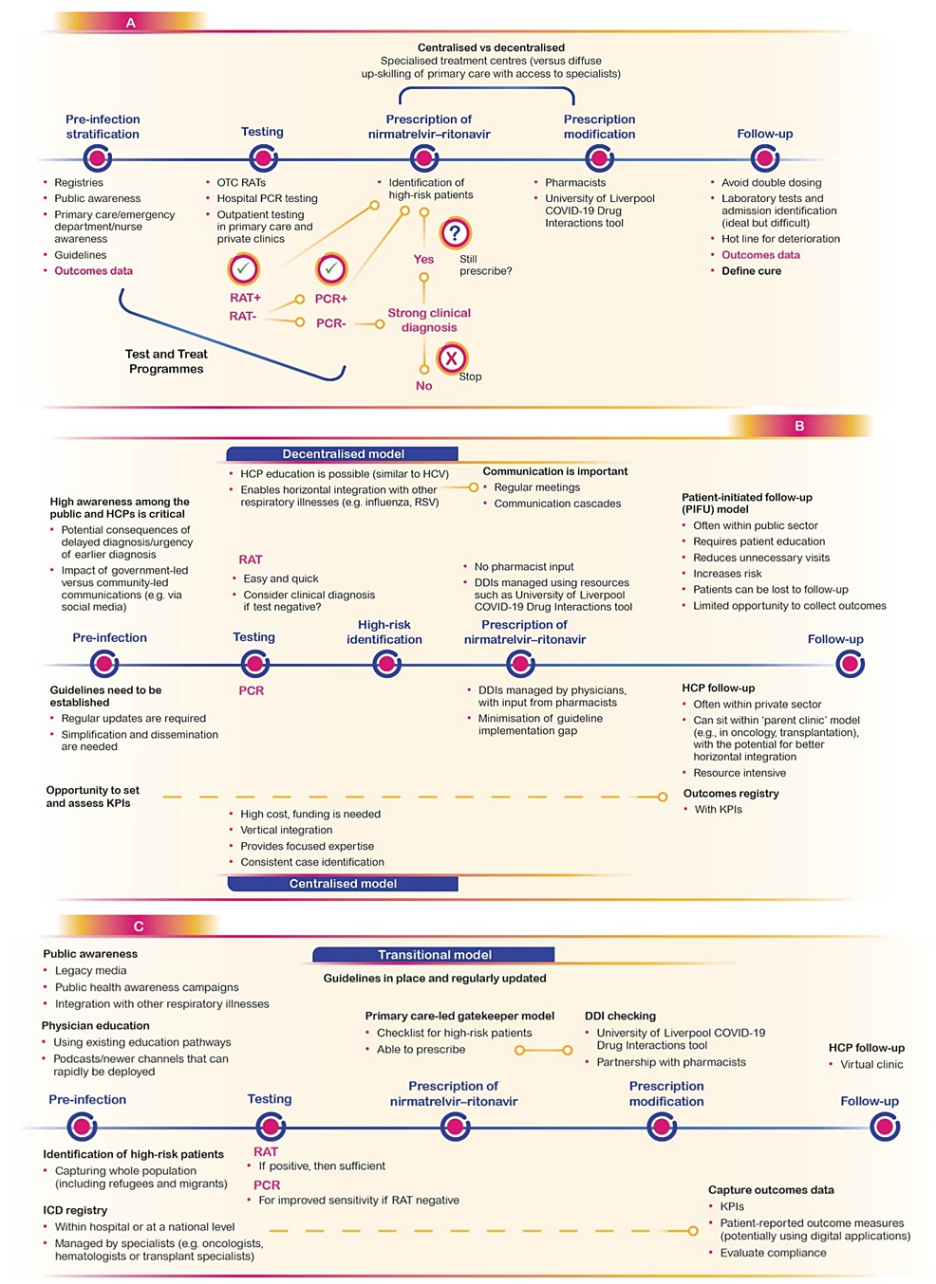
Proposed care pathways aimed at creating an enabling infrastructure for the management of COVID-19 patients at high risk of serious disease within different healthcare systems. A) Current infrastructure and variability between countries. B) Advantages and disadvantages of centralized and decentralized models. C) Proposed transitional model incorporating the advantages of both centralized and decentralized models. DDI - drug–drug interaction; HCV - hepatitis C virus; HCP - healthcare professional; ICD - International Classification of Diseases; KPIs - key performance indicators; OTC - over-the-counter; PCR - polymerase chain reaction; PIFU - patient-initiated follow-up; RAT - rapid antigen test; RSV - respiratory syncytial virus; UK - United Kingdom

The exact mechanism used to identify patients at high risk of progression to severe COVID-19 will depend on the healthcare system, for example, whether this process is centralized or decentralized (Figure 2). Participants proposed that integration with any existing treatment protocols in place for SARIs could be helpful in maximizing efficiency.

Decentralization of care from central tertiary hospitals into primary care clinics in the community has the potential to increase access to care, partly by increasing geographical coverage of care sites. A decentralized point-of-care framework would be appropriate for countries outside of North America and Western Europe, where many people live in rural areas and may have limited access to laboratory testing [26]. Decentralized care models for HIV have already shown how community-based interventions can improve clinical outcomes in LMICs [27,28].

A simple testing pathway was preferred by participants, to facilitate equity of access to testing in vulnerable populations, and test-and-treat programmes should be considered. In the US, the launch of a test-and-treat initiative contributed to an increase in dispensing rates for oral antiviral agents, accompanied by greater access to testing [29].

Although a positive RAT is considered sufficient to inform treatment, participants agreed that access to PCR testing is still necessary given that it can increase sensitivity in a RAT-negative patient or where RATs are not in routine use.

Participants did not reach a consensus on whether there is a need for separate specialist and general practice prescribing models for nirmatrelvir-ritonavir. Assessing the potential for DDIs is a critical part of prescribing nirmatrelvir-ritonavir. Consistently capturing the outcomes of treatment for every patient (potentially linked to key performance indicators) were considered essential for patient follow-up.

Future models of care will provide the opportunity to capture outcomes and measure the effectiveness of the care being provided. In many countries, the surveillance programs and registries that were established during the pandemic have been discontinued. For example, in South Africa, the national registry (DATCOV), which was commissioned in response to the COVID-19 pandemic, stopped collecting clinical data on patients admitted to hospitals across the country in the public and private sectors on 31 December 2022. It will be important to explore what future real-world data capture will be practical and most insightful for healthcare systems.

HCP and Pharmacist Preparedness

Following emergency use authorization for nirmatrelvir-ritonavir being issued by the US Food and Drug Administration (FDA), some physicians in the US have called for clearer guidance on its use [30]. A survey of primary care HCPs from the US suggested there is hesitancy in prescribing nirmatrelvir-ritonavir to older adults due to concerns around DDIs [31].

Because of the need for careful management of the potential for interactions with comedications, participants did not reach a consensus on whether PCPs should take the lead in identifying high-risk patients and prescribing nirmatrelvir-ritonavir. Those who were in favor consider DDIs as an aspect of prescribing for which appropriate training and education is necessary. Those not in favor believe that all high-risk patients should be managed by a specialist, including the prescribing of oral antivirals.

In the event that healthcare system regulations in individual countries allow for the prescribing of nirmatrelvir-ritonavir by non-physician HCPs (such as pharmacists or specialist nurses), participants generally agreed that expanded prescribing would have the benefit of increasing the likelihood of patients receiving treatment within the 5-day treatment window.

Nonetheless, there was broad consensus that additional education and training across disciplines (PCPs, specialists, and other HCPs) is essential to facilitate the identification of high-risk patients and allow expanded prescribing of antiviral therapy.

Patient and Public Awareness

The WHO recognizes that controlling the COVID-19 pandemic requires effective management of the associated epidemic of misinformation [32]. The quality of COVID-19 information available online is generally poor [33], and there is a need for HCPs to help combat the threat of COVID-19 misinformation [34]. Participants agreed that countering misinformation is important and will require the development (with multidisciplinary involvement) of evidence-based messaging and information that is simple, consistent, and coordinated across HCPs to ensure that patients receive correct information irrespective of the source.

Since some patients (and their caregivers or family members) do not necessarily self-identify as high-risk, participants agreed that education is needed on key risk factors for the general population. Developing a formal process to identify high-risk patients will require use of healthcare system data (specific to each country) and the involvement of specialists (given that in some healthcare systems many patients are managed by specialists).

Once developed, community-based awareness and education programs either to counter misinformation or to inform risk factor self-assessment should be delivered via a mix of media, including websites, television, 'influencers' (including religious leaders), and social media platforms (e.g., Facebook, Instagram, TikTok, Twitter, YouTube) to reach as many people as possible.

## Discussion

Expert perspectives and recommendations

We have identified notable variations in approaches to the management of high-risk patients with COVID-19 between countries across the Middle East, Africa, and Eastern Europe. In 2020, many countries put in place vertical solutions that made the best use of the resources available within their healthcare system and wider society to address the challenges and priorities of their population, leading to significant innovation in a short period of time. Each individual country’s pathway is associated with specific advantages and disadvantages within its healthcare setting, but no one approach reflects the ideal best practice in entirety. However, through bringing together diverse perspectives and experiences from across the global scientific community, there is an opportunity to align on optimal care pathways that allow us to address future COVID-19 waves more effectively. Indeed, international working groups could play a key role in ensuring the resilience of global healthcare systems, enabling them to provide optimal care for patients at high risk in future pandemics.

A striking insight gained from a number of countries is that the main entry point for care is often not a physician. In some countries, the care pathway for high-risk patients with symptoms of COVID-19 is effectively managed by nurses or other technical staff with input from specialized HCPs at key decision nodes, such as managing DDIs. Assessing the needs of patients, healthcare contributors beyond physicians can play a key role in counseling high-risk patients, including sharing important information and disseminating educational messages. It can be anticipated that the broader multidisciplinary healthcare team will play a key role in managing future pandemics, and their voices should also be heard in the process of future-proofing care pathways.

WHO technical guidance [35] served as a foundational resource during the emergence of the pandemic. Moving forward, more focus on international guidelines on pertinent clinical questions (such as who and where to test, where symptomatic patients should present, how cases should be escalated, how to enable best practice care, and how to deliver effective public awareness campaigns) would be highly valuable globally.

Participants provided several recommendations. They noted that even though primary care is often the first point of contact for high-risk patients, it remains uncertain whether primary care providers should play a larger role in their treatment. They stressed that increased education and training across disciplines are essential to help identify high-risk patients and expand the prescribing of antiviral therapy. Potential formats and content for educational activities suggested by participants included training strategies for primary care providers, potentially with infectious disease specialists, and the creation and maintenance of a communication channel where primary care providers can share experiences and discuss patient cases to feel more confident in managing high-risk patients, including prescribing antiviral medicines. Programs aimed at increasing the awareness and knowledge of patients were also highlighted by participants as an important educational strategy.

They suggested that improving the uptake of antiviral therapy in countries with high HIV rates requires more clinical evidence, including results from clinical trials and real-world evidence, on the use of nirmatrelvir-ritonavir in patients with HIV. They also expressed concerns about the management of comedications and potential interactions with nirmatrelvir-ritonavir, but suggested that prescribers can gain confidence through education, the use of online tools, and the support of pharmacists. Although participants agreed that pharmacists should not usually be the ones to prescribe nirmatrelvir-ritonavir; they emphasized that pharmacists have a critical role to play in identifying clinically important potential interactions and recommending dosage adjustments as needed.

Our study has a number of limitations. The snowball sampling technique has a potential sampling error owing to participants being more likely to refer people who share their characteristics or experiences. It is a qualitative analysis based on the retrospective perspectives of a small sample of HCPs. Additional quantitative insights from a bigger sample would add to our understanding of current practices and how we can share experiences to improve patient care and, ultimately, understand how to approach future pandemics/COVID-19 waves.

Limitations

Our study has a number of limitations. The snowball sampling technique has a potential sampling error owing to participants being more likely to refer people who share their characteristics or experiences. It is a qualitative analysis based on the retrospective perspectives of a small sample of HCPs. Additional quantitative insights from a bigger sample would add to our understanding of current practices and how we can share experiences to improve patient care and, ultimately, understand how to approach future pandemics/COVID-19 waves.

## Conclusions

HCPs from across the Middle East, Africa, and Eastern Europe came together at a regional summit in February 2023 to share perspectives and practice insights from their respective countries with the goal of providing a more complete global picture of the therapeutic management of patients at high risk of serious COVID-19 disease in the community.

Participants noted that differences between countries in the care pathway for high-risk patients are due to variations in local treatment practices, healthcare system structures, and resources. They warned that patient hesitancy can lead to late presentations, delayed treatment, and potentially more severe symptoms. They proposed that targeted awareness and education initiatives could help patients identify risk factors for hospitalization, reduce their reluctance to undergo testing for COVID-19, and counter misinformation about the virus. It was recommended that healthcare providers facilitate access to prompt testing and treatment and provide proactive patient counseling to ensure that high-risk patients understand their risk and its implications.
